# Prognostic Factors of Papillary and Follicular Carcinomas in Japan Based on Data of Kuma Hospital

**DOI:** 10.1155/2012/973497

**Published:** 2011-09-29

**Authors:** Yasuhiro Ito, Akira Miyauchi

**Affiliations:** Department of Surgery, Kuma Hospital, 8-2-35, Shimoyamate-dori, Chuo-ku, Kobe 650-0011, Japan

## Abstract

There are some important prognostic factors for papillary thyroid carcinoma (PTC) and follicular thyroid carcinoma (FTC). In this paper, clinicopathological features significantly affecting patient prognosis are described based on our data as well as others. Distant metastasis at diagnosis is the most important prognostic factor for both PTC and FTC. Other than that, preoperative and intraoperative findings are important to evaluate the biological behavior of PTC. Extrathyroid extension, large lymph-node metastasis, and extranodal tumor extension that can be evaluated preoperatively or intraoperatively are significant prognostic factors for PTC patients. In contrast, pathological findings are important not only for diagnosis of FTC, but also for the evaluation of its biological character. Grade of invasiveness (minimally or widely invasive) and degree of differentiation (well differentiated or including a poorly differentiated component) greatly affect the prognosis of FTC patients.

## 1. Introduction

There are two histological types of thyroid carcinoma arising from follicular cells, which are papillary carcinoma (PTC) and follicular carcinoma (FTC). These two histological types are also called differentiated carcinoma (DTC) and analyzed as a single group for clinical studies investigating prognostic factors and prognosis of patients. However, biological behaviors of these two carcinomas significantly differ. PTC frequently metastasizes to the regional lymph nodes and can show a high incidence of significant extrathyroid extension to adjacent organs. In contrast, it is comparably rare for FTC to show these events, but FTC more frequently metastasizes to distant organs such as the lung, bone, and brain than PTC. Therefore, analyses of prognostic factors and prognosis of patients should be performed separately for PTC and FTC. Generally, PTC and FTC are indolent diseases and show good prognoses of patients, but when the lesion dedifferentiates, becoming undifferentiated carcinoma (anaplastic carcinoma), the prognosis of patients turned to be extremely poor. The mechanism of dedifferentiation remains unknown although the *p53* gene mutation is frequently observed in UC [1].

 In the 1980s, a pathological entity of poorly differentiated carcinoma (PDC) was proposed by Sakamoto et al. [2] and Carcangiu et al. [3]. PDC implies thyroid carcinoma of intermediate type between DTC and UC. It was adopted as an independent histopathological category from PTC and FTC in the WHO classification [4]. However, as described below, there are three kinds of diagnostic criteria for PDC defined in General Rules for the Description of Thyroid Cancer established by Japanese Society of Thyroid Surgeons (JSTS) [5], in WHO classification [4], and in the Turin proposal [6], which generates confusion among pathologists and clinical physicians. 

 In this paper, we present the prognosis and prognostic value of PTC and FTC together with PDC based on data from Kuma Hospital as well as search results of studies from other institutes. Since the traditional Japanese diet is iodine-rich, the results may somewhat differ from those in Western countries.

## 2. Prognostic Factors of PTC

To date, several prognostic factors have been identified for PTC. These factors can be divided into 4 categories, backgrounds of patients, factors based on preoperative, and intraoperative and postoperative evaluations. Backgrounds of patients include age, gender, and family history. Preoperative evaluation is mainly performed by imaging studies. Of these, ultrasonography is the most useful tool to detect and evaluate primary lesions [7] and regional lymph node metastasis except for metastasis to the mediastinal and retropharyngeal nodes. In order to diagnose metastasis to the upper mediastinal node and retropharyngeal node, other imaging studies such as CT scan, MRI, and PET-CT are useful. Ultrasonography cannot only detect thyroid nodules, but also qualitatively diagnose these nodes based on the findings. Our institution diagnoses whether thyroid nodules are malignant or benign based on our own ultrasonographic grading system [7]. In association with ultrasonography-guided fine-needle aspiration biopsy (FNAB), minute thyroid carcinoma, especially PTC, can be diagnosed. Furthermore, size, location, and multiplicity of primary lesions can be evaluated on ultrasonography. Lymph node metastasis can also be diagnosed based on ultrasonographic criteria. Among the systems available, the criteria proposed by Antonelli et al. [8] are representative: maximal diameter >1 cm, clear hypoechoic pattern, and rounded (shorter/longer diameter ratio > 0.7) or bulging shape with increased anteroposterior diameter. These criteria are almost the same as those used in our institution except for maximal diameter >1 cm. Ultrasonography-guided FNAB and thyroglobulin measurement on wash-out of needles used for FNAB [9] could be a great help to diagnose whether the node is metastatic or reactive. Especially, it is useful to diagnose whether clinical node metastasis in the lateral compartment (level II–IV) is present and to decide whether therapeutic modified radical neck dissection (MND) should be performed. 

 Intraoperative evaluation is based on findings during surgery, including extrathyroid extension and extranodal tumor extension to adjacent organs. The degree of extension, that is, to where and how the tumor extends, significantly affects the prognosis of patients as described later.

 Postoperative evaluation includes findings based on pathological and molecular examinations. There are various histological types in PTC, most of which are diagnosed on pathological examination. Molecular examination includes, for example, *BRAF* mutation analysis.

### 2.1. Prognostic Factors Related to Patient Backgrounds 

#### 2.1.1. Patient Age

**Figure 1:**
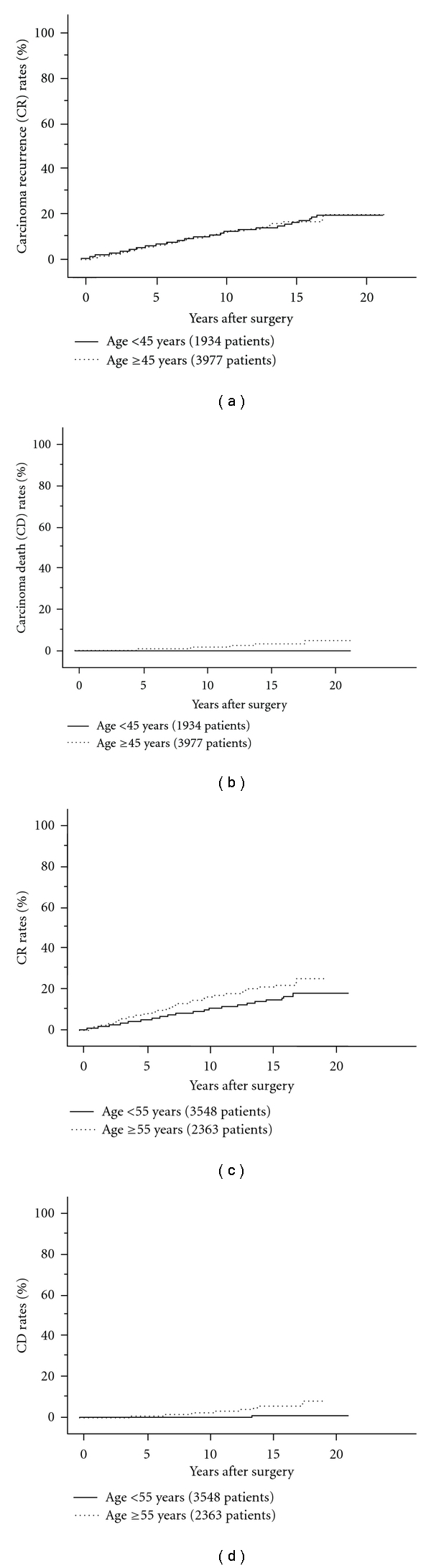
(a) Carcinoma recurrence (CR) rate of PTC patients aged 45 years or older and those younger than 45 years. (b) Carcinoma death (CD) rates of PTC patients aged 45 years or older and those younger than 45 years. (c) CR rates of PTC patients aged 55 years or older and those younger than 45 years. (d) CD rates of PTC patients aged 55 years or older and those younger than 45 years.

Patient age is an important background factor for predicting prognoses. Several classification systems have adopted age as a prominent factor in deciding whether carcinoma should be considered high risk. For example, AMES set the cutoff age at 41 for males and 51 for females to discriminate between high-risk and low-risk patients [10]. UICC TNM classification [11] and CIH classification [12] set the cutoff age at 45 years and 50 years, respectively. MACIS is a scoring system that also includes patient age as a very significant factor [13]. In our setting series, the cutoff age at 45 years affected the cause-specific survival (CSS) of patients to some extent, but its prognostic impact for disease-free survival (DFS) was weak [14] (Figures 1(a) and 1(b)). Currently, we set the cutoff age at 55 years, because this most significantly reflected patient DFS and CSS in our series (Figures 1(c) and 1(d)). On multivariate study, age at 55 years or older was the most significant prognostic factor for CSS, except for distant metastasis at surgery [15].

#### 2.1.2. Gender

Previous studies showed discrepant findings regarding the prognostic value of gender [16–18]. In our analysis of 6015 PTC patients, male gender was an independent but moderate prognostic factor for DFS and CSS [15]. 

As indicated above, age and gender each independently affect patient prognosis. However, our recent data indicate that the significance of other prognostic factors varied according to patient age and gender when analyzed in four subsets of patients; older female, older male, younger female and younger male. This issue will later be described briefly as an addendum.

#### 2.1.3. Family History

**Figure 2:**
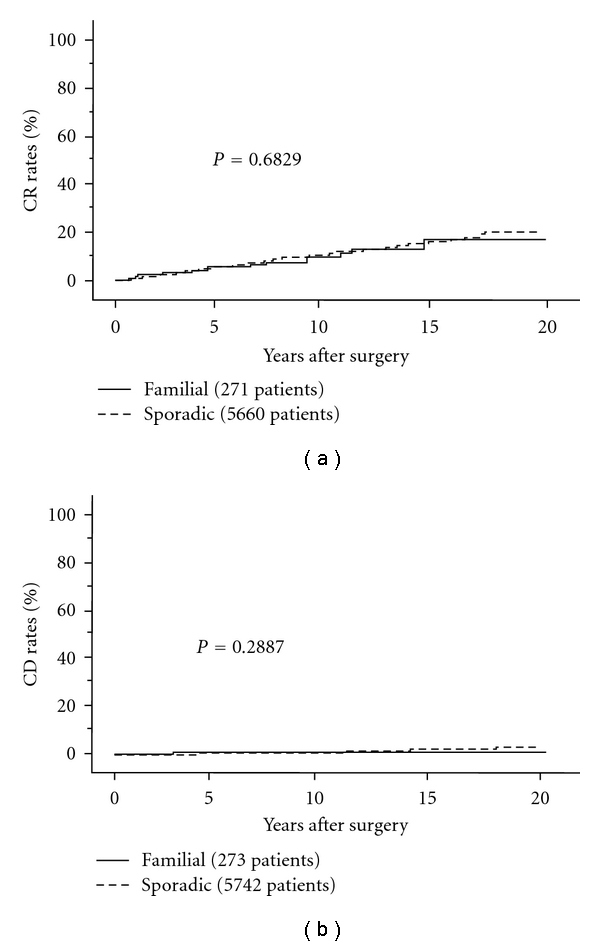
(a) CR rates of familial and sporadic PTC patients.

PTC and FTC are generally considered sporadic with the exception that these are lesions also associated with rare inherited diseases such as familial adenomatous polyposis, Gardner syndrome, and Cowden disease [19, 20]. However, case reports of DTC in monozygotic twins and in a mother and son were published in 1955 and 1975, respectively, [21, 22]. More recently, population studies showed that the risk of DTC was elevated in individuals with a first-degree relative having DTC [23, 24]. Although the genes that cause these lesions have not been identified, a definition of familial nonmedullary thyroid carcinoma (FNMTC) has been established as patients with DTC having one or more affected persons among their first-degree of relatives [25]. It remains controversial whether FNMTC shows a different prognosis from sporadic DTC [26–30]. In Japan, there are two studies investigating this issue in a large series of patients [31, 32]. One study showed that FNMTC was more likely to recur, but its CSS did not differ from that of sporadic DTC [31]. The other study demonstrated that DFS and CSS of familial PTC did not differ from sporadic PTC [32] (Figures 2(a) and 2(b)). Interestingly, the incidences of FNMTC in the two studies were similar at about 5%. Currently, we have no evidence that familial PTC shows a significantly worse DFS than sporadic PTC or that the therapeutic strategy should be changed. However, familial PTC is more likely to be multiple, and recurrence rate to the remnant thyroid, if total thyroidectomy is not performed, is higher than that in patients with sporadic lesions (5% versus 1%) [32]. Therefore, total thyroidectomy is recommended for familial PTC patients regardless of carcinoma stage.

### 2.2. Prognostic Factors Predominantly Based on Preoperative Evaluation

#### 2.2.1. Tumor Size

Tumor size was adopted as a factor discriminating high-risk patients from others in various classification systems such as UICC classification [11], MACIS scoring system [13], and AMES [10]. In UICC TNM classification, there are two cutoffs, 2 cm and 4 cm (T1 for 2 cm or less, T2 for 2.1–4 cm, and T3 for larger than 4 cm) [11]. In AMES, 5 cm is a cutoff between high-risk and low-risk patients [10]. 

 Recently, an observation trial for PTC measuring 1 cm or less (microcarcinoma) without any high-risk features such as clinical lymph node and/or distant metastasis has been performed in some institutions in Japan [33–37]. In our data, only about 7% of low-risk microcarcinoma significantly enlarged during followup once or twice per year for 5 years, and none of these patients showed recurrence or died of carcinoma after surgery at the appearance of progression signs such as tumor enlargement and newly detected lymph node metastasis. In Japan, therefore, cutoff at 1 cm was considered important and General Rules for the Description of Thyroid Cancer established by JSTS further divided T1 into two categories, T1a (1 cm or less) and T1b (1.1–2 cm) [5]. Previous autopsy studies demonstrated that small thyroid carcinoma was detected with a significantly high incidence [38–40]. Furthermore, thyroid carcinomas were detected in 3.5% of otherwise healthy Japanese women aged 30 years or older using ultrasonography and ultrasonography-guided FNAB [41]. Thus, it is suggested that most small thyroid carcinomas remain latent and do not or only very slowly grow. The successful results of an observation trial of microcarcinoma indicated that the above should, therefore, be reasonable.

**Figure 3:**
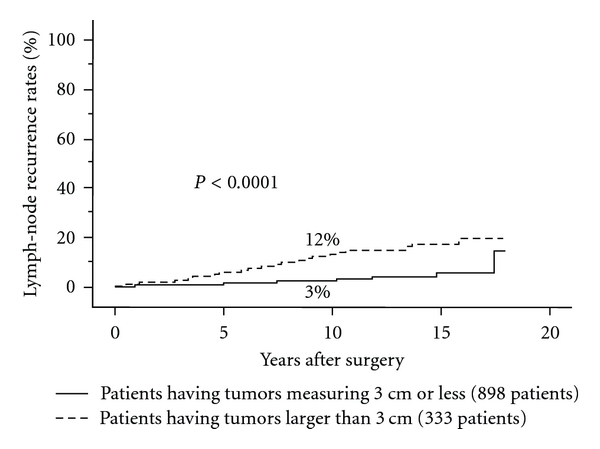
Lymph-node recurrence rates of PTC patients having tumors larger than 3 cm and those having tumors measuring 3 cm or less.

 In contrast, in our data based on a series of patients treated in our institution, tumor size larger than 4 cm significantly affected DFS and CSS of PTC patients on multivariate analysis [15]. Therefore, careful and extensive surgery and postoperative followup are recommended for PTC patients larger than 4 cm even though there are no other high-risk features. Furthermore, 10-year lymph-node recurrence rates of N0 or N1a PTC patients having tumors larger than 3 cm were higher at 13% than that of those measuring 3 cm or less, which was 3% even though patients underwent central node dissection and prophylactic MND [42] (Figure 3). If prophylactic MND were not performed, lymph-node recurrence rate would be even higher for N0 or N1a PTC larger than 3 cm. Based on these data, our institution performs prophylactic MND for N0 or N1a PTC with a tumor larger than 3 cm. Sugitani et al. also showed that PTC larger than 4 cm should be indicated for prophylactic MND [43]. 

#### 2.2.2. Multiplicity of Primary Lesions

PTC is frequently multiple. We previously showed that lateral node metastasis is more frequently detected in multiple microcarcinomas than in solitary microcarcinomas [34]. It is, therefore, suggested that multiplicity reflects the aggressive behavior of PTC to some extent. However, in our study analyzing over 6000 patients with PTC, multiplicity was not an independent prognostic factor on multivariate analysis [14]. 

**Figure 4:**
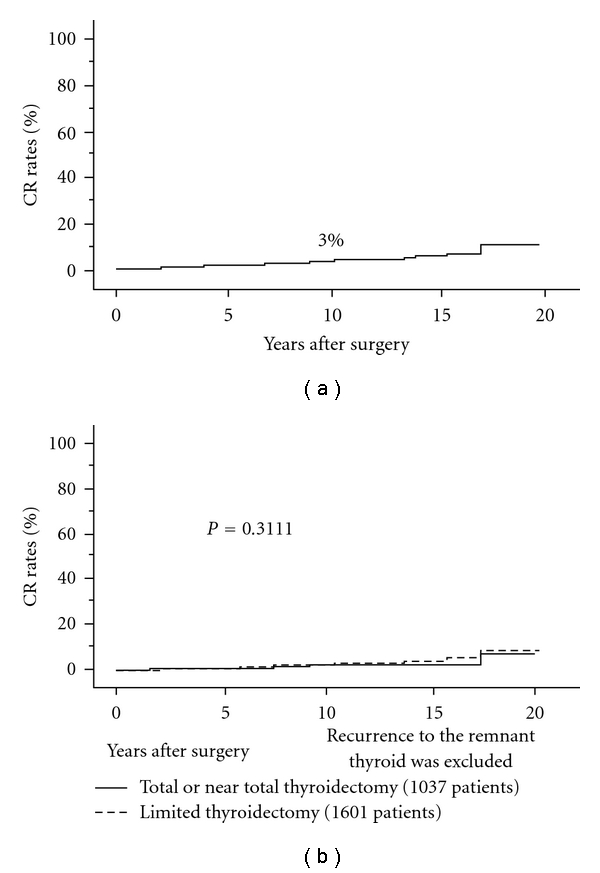
(a) CR rates of solitary T1N0M0 PTC patients. (b) CR rates of solitary T1N0M0 PTC patients who underwent total or near total thyroidectomy and those who underwent limited thyroidectomy excluding recurrence to the remnant thyroid. Recurrence to the remnant thyroid is ignored.

 There is a limitation in diagnosing multiplicity of PTC on ultrasonography. Our data on microcarcinoma demonstrated that the diagnostic accuracies of ultrasonography for multiplicity were 53% for sensitivity and 85% for specificity [14]. This indicates that preoperative evaluation on ultrasonography often overlooks multiplicity of carcinoma, which is one of the reasons for almost routine recommendation of total thyroidectomy for PTC patients. However, our institution generally performs hemithyroidectomy for patients with PTC measuring 2 cm or smaller (T1 in UICC TNM classification) diagnosed as solitary on ultrasonography, unless they have clinical lymph node or distant metastasis or family histories. The 10-year carcinoma recurrence rate was only 5% for these patients (Figure 4(a)). The risk of recurrence to the remnant thyroid was only 1% for patients who underwent limited thyroidectomy, and, if this risk is ignored, the extent of thyroidectomy did not affect patient prognosis (Figure 4(b)) [44]. Even though tiny PTC lesions remain undissected, these lesions are generally harmless for such low-risk patients. Therefore, we currently conclude that total thyroidectomy is not mandatory for solitary T1N0M0 patients.

#### 2.2.3. Clinical Lymph Node Metastasis (N)

Lymph node metastasis is a very common event and recognized as one of the most important prognostic factors. However, prominent classification systems such as AMES [10] and MACIS [13] do not adopt lymph-node metastasis as a prognostic factor. This may be possibly because these systems were based on a series of patients who underwent surgery before the establishment of routine ultrasonography as a preoperative imaging study for accurate evaluation of lymph-node metastasis. 

 At present, lymph-node metastasis can be preoperatively evaluated on imaging studies, and, as indicated above, ultrasonography is the most useful tool for this purpose. Node metastasis detected on preoperative imaging studies is called clinical lymph node metastasis (N), which is discussed in this section. Similar to multiplicity, ultrasonography often overlooks lymph-node metastasis, which can only be diagnosed on postoperative pathological examination in a large number of cases, as described later.

 Evaluation of clinical node metastasis is very important, and it was divided into two categories in UICC TNM classification [11]: N1a, central node metastasis and N1b, metastasis to the lateral or mediastinal compartment. In this classification, N1b is upgraded compared to N1a and N1b patients are further upstaged if they are aged 45 years or older. 

**Figure 5:**
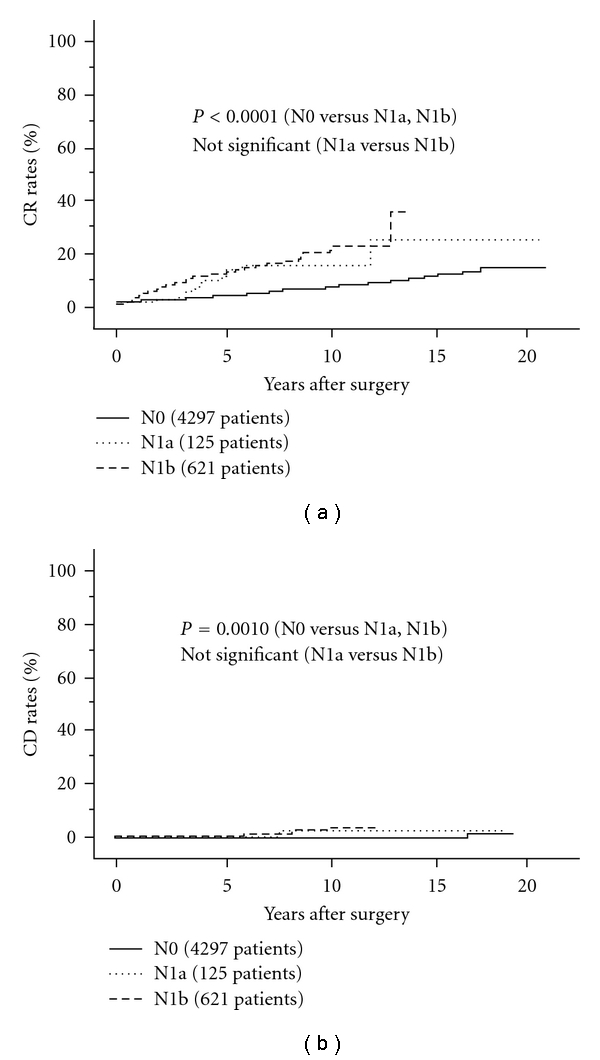
CR rates of N0, N1a, and N1b PTC patients without extrathyroid extension.

 Indeed, N has an important prognostic impact. We previously showed that N1b was an independent prognostic factor affecting both DFS and CSS [33, 45–49]. However, more recently, we also demonstrated that in the subset of patients without significant extrathyroid extension, prognosis of patients with N1b did not much differ from that of N1a patients although it was significantly worse than N0 patients [50] (Figures 5(a) and 5(b)). Therefore, it remains unclear whether it is appropriate to upgrade N1b compared with N1a. Importantly, CIH classification adopted lymph-node metastasis measuring 3 cm or larger as a factor of high risk [11]. We analyzed this issue in our series and obtained similar results [50]. In the same series, we also found that extranodal tumor extension to adjacent organs requiring the dissection of these organs that was predominantly evaluated on intraoperative findings had a significant prognostic value especially for CSS as indicated below [14, 50, 51].

**Figure 6:**
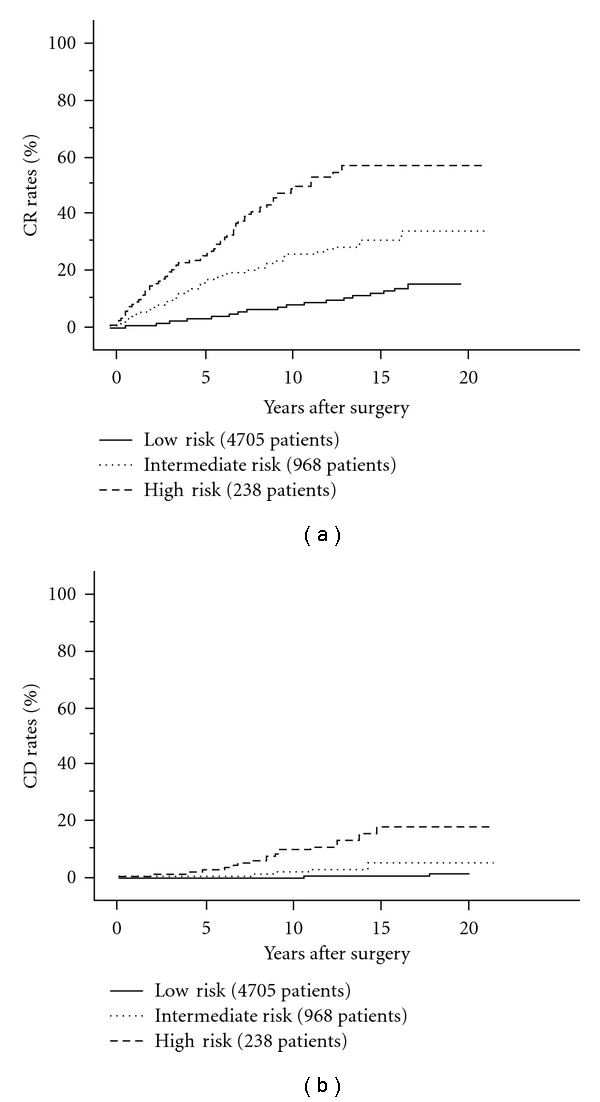
CR rates of PTC patients with low risk, intermediate risk, and high risk for lymph-node metastasis.

 Taken together, it is currently considered that the prognostic impact of clinical node metastasis can be divided into three categories: (1) clinical node metastasis measuring 3 cm or larger or showing extranodal tumor extension on intraoperative findings (high risk), (2) clinical node metastasis smaller than 3 cm without extranodal tumor extension (intermediate risk), and (3) no clinical node metastasis (low-risk). Figures 6(a) and 6(b) indicate carcinoma recurrence rate and carcinoma death rate of three categories for carcinoma recurrence rate and carcinoma death rate. 

#### 2.2.4. Distant Metastasis at Surgery (M1)

Although rarer than FTC, PTC can metastasize not only to regional lymph nodes, but also to distant organs such as the lung, bone, and brain. Distant metastasis at surgery can be detected on imaging studies such as CT scan and PET-CT and also on postoperative radioactive iodine (RAI) ablation or whole body scan. 

**Figure 7:**
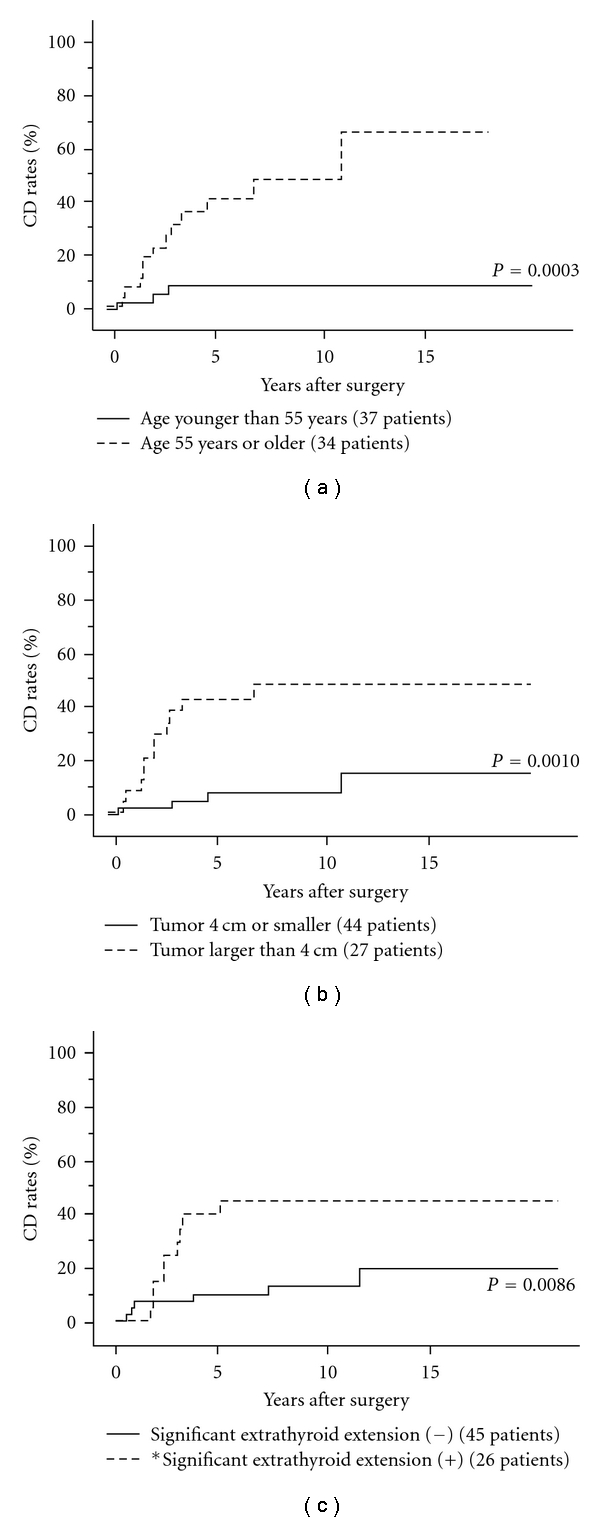
(a) CD rates of M1 patients with PTC aged 55 years or older and those aged younger than 55 years. (b) CD rates of M1 patients with PTC having tumor 4 cm or smaller and those having tumor larger than 4 cm. (c) CD rates of M1 PTC patients with and without extrathyroid extension. *Except for recurrent laryngeal nerve and cricothyroid or inferior constrictor muscle.

 There are no doubts that distant metastasis at surgery is one of the most important prognostic factors for CSS of patients [14]. However, prognosis of M1 patients differs according to other clinicopathological features of the patient. Many previous studies analyzed M1 patients and patients showing distant recurrence during postoperative followup as a single group and/or analyzed PTC and FTC as DTC in a single group [52–55]. In our opinion, these should be separately analyzed as described in the Introduction. In the subset of M1 patients with PTC in our series, M1 is directly linked to other clinicopathological features such as gender, tumor size, extrathyroid extension, and N factor, indicating that distant metastasis at diagnosis will more likely be found in PTC showing aggressive behavior [56]. Tumor larger than 4 cm, aged 55 years or older (at the time of initial surgery) and extrathyroid extension were independent prognostic factors for CSS of M1 PTC patients (Figures 7(a), 7(b), and 7(c)). Distant metastasis to organs other than the lung also reflected worse CSS of patients on univariate analysis. Furthermore, although there was no significant difference, CSS of M1 patients whose distant metastases were refractory to RAI therapy was also adverse. 

 Taken together, the prognosis of M1 patients depends on clinicopathological features of the primary lesion.

### 2.3. Prognostic Factors Predominantly Based on Intraoperative Findings

#### 2.3.1. Extrathyroid Extension

Extrathyroid extension has been adopted in various classification systems [10–13]. In the UICC TNM classification system, there are two grades of extrathyroid extension [11]. Extension to perithyroid tissue and sternothyroid muscle was graded as T3 (minimal extension), and extension to other adjacent organs such as the recurrent laryngeal nerve, esophagus, trachea, sternohyoid muscle, and jugular vein was graded as T4 (massive or significant extension). However, this classification has some limitations. Firstly, this classification system is established for preoperative evaluation. However, it is significantly difficult to accurately evaluate extrathyroid extension based on preoperative evaluation unless recurrent laryngeal nerve paralysis due to carcinoma invasion and apparent intratracheal extension on CT scan or MRI can be detected. Most extrathyroid extensions are found on intraoperative findings, indicating that T classification based on preoperative findings is not appropriate. It is almost impossible to detect T3 based on preoperative findings and evaluation of T4 on preoperative findings is also much more inaccurate than that on intraoperative findings. Secondly, our previous studies showed that T3 did not affect patient prognosis either on intraoperative or pathological evaluation [57, 58]. In UICC TNM classification, tumors measuring 4 cm or less are upgraded to T3 if they showed minimal extension, but we proposed that such an upgrade should be abolished based on our data. In our institution, only significant extrathyroid extension on intraoperative findings is adopted as a factor indicating aggressive behavior.

 Our recent study showed that the significance of extrathyroid extension is not uniform but rather size-dependent. Prognostic significance of extrathyroid extension was less than clinical lateral node metastasis (N1b) for PTC measuring 3 cm or less, it was reversed in PTC larger than 3 cm [59]. In the intraoperative staging system that we established by revising the UICC TNM staging system, extrathyroid extension of tumor larger than 2 cm was regarded as a sign of high risk and that of a tumor 2 cm or smaller was a sign of intermediate risk [14].

 We currently conclude that extrathyroid extension should be evaluated on intraoperative findings and that minimal extension to perithyroid tissue and the sternothyroid muscle should not be considered significant. Significant extension on intraoperative evaluation is an important factor predicting a worse prognosis for patients with PTC, especially those with a large tumor.

#### 2.3.2. Extranodal Tumor Extension

Prognostic significance of extranodal tumor extension has been investigated by several groups including ours [14, 50, 51, 60–62]. Yamashita et al. showed that patients with pathological extranodal tumor extension were more likely to show distant recurrence [61]. In our series, as indicated above, extranodal tumor extension requiring resection of adjacent organs showed a worse prognosis, especially for CSS as indicated above [14, 50, 51]. It is strongly suggested that PTC with extranodal tumor extension is high risk and has a high potential to show a dire prognosis.

### 2.4. Prognostic Factors Based on Postoperative Findings

#### 2.4.1. Pathological Lymph-Node Metastasis

PTC frequently metastasizes to the regional lymph nodes [63–65]. As indicated above, clinical lymph-node metastasis detected on preoperative imaging studies is a significant prognostic factor, and especially, large metastatic node has a very strong prognostic impact on both DFS and CSS of PTC patients. However, diagnostic accuracy of ultrasonography for lymph-node metastasis is actually not very high. Our previous study showed that the positive predictive value (PPV) and specificity of ultrasonography for central node metastasis were 92% and 98%, respectively, but the negative predictive value (NPV) and sensitivity were only 37% and 12%, respectively, [15]. For lateral node metastasis, PPV and specificity were 95% and 97%, respectively, and NPV and sensitivity were 43% and 29%, respectively, [15]. Diagnostic accuracy of ultrasonography for lateral node metastasis is a little better than that for central node metastasis, but these findings indicate that small and latent metastasis to the regional lymph nodes is frequently overlooked on ultrasonography. However, according to our data, such latent metastases do not markedly affect patient prognosis. Pathological and latent node metastases increase the rate of carcinoma recurrence to some extent but do not affect CSS of patients [32, 45]. In conclusion, lymph-node metastasis that can be diagnosed only on pathological examination is a moderate factor only for PTC recurrence.

#### 2.4.2. Histological Variants

**Figure 8:**
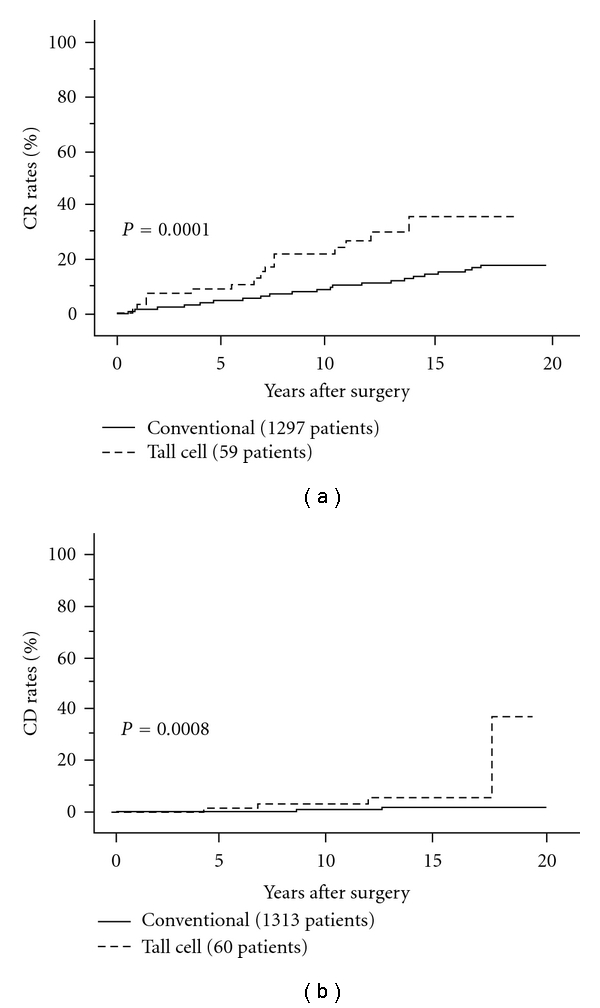
CR rates of tall cell variant and conventional PTC.

Many histological variants of PTC have been adopted in the WHO classification [4].  Table 1 summarizes the prevalence of histological variants in our series of 1521 PTC patients [66]. Follicular variant was the most common variant, which accounted for 7%. Follicular variant was reported to show aggressive behavior [67–69], but in our series of Japanese patients, prognosis did not differ from that of conventional PTC [66]. Tall cell variant is a typical variant showing an aggressive behavior [70–72] (Figures 8(a) and 8(b)). This accounted for 4% of our series of PTC patients [73]. Interestingly, the incidence of clinicopathological features reflecting poor prognosis such as gender, clinical lymph-node metastasis, and extrathyroid extension did not differ between tall cell variant and others although the average age of patients with tall cell variant was slightly higher than that of other patients. However, this histology independently affected DFS and CSS of PTC patients on multivariate analysis [73]. Oncocytic variant accounted for 2% of PTC and most of them were diagnosed as having Warthin-like tumor showing abundant chronic inflammatory cells that are associated with chronic thyroiditis. Previous studies showed that this variant generally had a mild character [74–76], which was identical to our findings, because none of the patients with this oncocytic variant died of carcinoma in our study [66].

 There are some more important variants of which prevalence is lower than those indicated above. Columnar cell variant is now classified as an independent entity as columnar cell carcinoma [4]. This carcinoma accounted only for 0.4%, but as much as 60% of patients showed carcinoma recurrence, indicating that this histologic type is a sign of significantly aggressive behavior [66]. Regarding the biological behavior and prognosis of the diffuse sclerosing variant, previous studies showed discrepant findings [77–79]. In our study, the diffuse sclerosing variant frequently showed multiple clinical node metastases and was more likely to show PTC recurrence, but the CSS of patients did not differ from that of conventional PTC [80]. Macrofollicular variant could be diagnosed as multinodular goiter in the past in high incidences [81, 82]. In our study, it shows an indolent character, similar to reports from Western countries [83]. Cribriform morular variant is mostly a hereditary disease caused by the *APC* gene mutations associated with colonic polyposis or colon carcinoma [84]. This variant is multicentric and total thyroidectomy is mandatory regardless of carcinoma size and lymph-node status, but the prognosis of patients is generally excellent [85].

 Although not adopted in the WHO classification, encapsulated, PTC generally shows a better prognosis than conventional PTC. This type is encapsulated and there is no extrathyroid extension, which may be the reason for excellent prognosis. The incidence of lymph-node metastasis is also lower than that in conventional PTC [86, 87].

#### 2.4.3. Involvement of PDC Components

As indicated in Section 1, three criteria for PDC have been proposed. There are three growth patterns of PDC, solid, trabecular, and insular growth patterns, which are designated as PDC components. In order to diagnose PDC using the WHO classification, PDC components should occupy in the majority of the tumor [4]. However, in the JSTS criteria [5], carcinoma with only a small portion of PDC components is diagnosed as PDC and discriminated from PTC or FTC. In the criteria for PDC in the Turin proposal [6], the absence of nuclear features of PTC and the presence of convoluted nuclei, mitotic activity (3 × 10 HPF), or tumor necrosis were adopted in addition to the presence of a PDC component. 

 At present, PDC is considered as an independent entity from PTC and FTC in WHO classification [4] and JSTS [5], and it may be better to describe PDC in an independent chapter. However, the diagnostic criteria for PDC have not yet been consolidated. Thus, in this paper, the involvement of PDC components is regarded as one of the pathological features of both PTC and FTC.

 Actually, few studies have been published regarding the prevalence of PDC based on all three criteria in one series of patients. In our series, the prevalences of PDC based on JSTS guidelines, the WHO classification, and the Turin proposal were 11%, 0.8%, and 0.3%, respectively, in our PTC series [73]. This suggests that, in most cases, PDC components are present only in a small portion of carcinoma lesions. In our series, the 10-year DFS rates of PTC with and without PDC components were 88.8% and 77.0%, respectively, and the CSS rates were 94.2% and 98.4%, respectively. On multivariate analysis, the presence of PDC components independently reflected DFS but not CSS of patients. Based on these findings, it does not seem appropriate for PTC with limited PDC components (PDC on JSTS) to be classified as an independent entity. A recent study from Japan, however, demonstrated that DTC having a PDC component comprising 10% or more of the carcinoma lesion had a dire prognosis [88] and publications from other institutions are expected to elucidate this issue. In our series, the 10-year DFS and CSS rates of patients with PDC according to the WHO classification were 54% and 80%, respectively, and it is considered reasonable to regard this carcinoma as an independent entity. Patient with PDC according to the Turin proposal accounted for only 0.3% in this series, and the 10-year DFS and CSS were 25% and 60%, respectively. This carcinoma has a significantly more aggressive character, but in Japan, the incidence is very small compared with that in Europe and the United States [89, 90].

#### 2.4.4. Cell Proliferating Activity

Cell proliferating activity is regarded as an important biological behavior of human carcinoma. There are some markers for evaluation of cell proliferating activity, mitotic figure count (MFC), proliferating cell nuclear antigen (PCNA), and Ki-67. In PTC, however, cell proliferating activity is generally low. MFC that is evaluated on hematoxylin and eosin-stained tissue sections reflects only the index of mitotic cells, indicating that it is difficult to evaluate in PTC [91]. PCNA and Ki-67 can be evaluated by immunohistochemistry. The former is a cell cycle-related protein showing an elevated expression level in cells exclusively in the G1-S phase [92]. Ki-67 is a protein expressed in the cell nuclei in all cells except those in the G0 phase [93]. Therefore, labeling index (LI) of PCNA should be smaller than Ki-67 LI, but previous studies showed that PCNA LI was generally higher than Ki-67 LI [94, 95]. Furthermore, PCNA LI were reported not to correlate with Ki-67 LI or MFC [94], indicating that PCNA is an unreliable marker for evaluation of cell proliferating activity.

**Figure 9:**
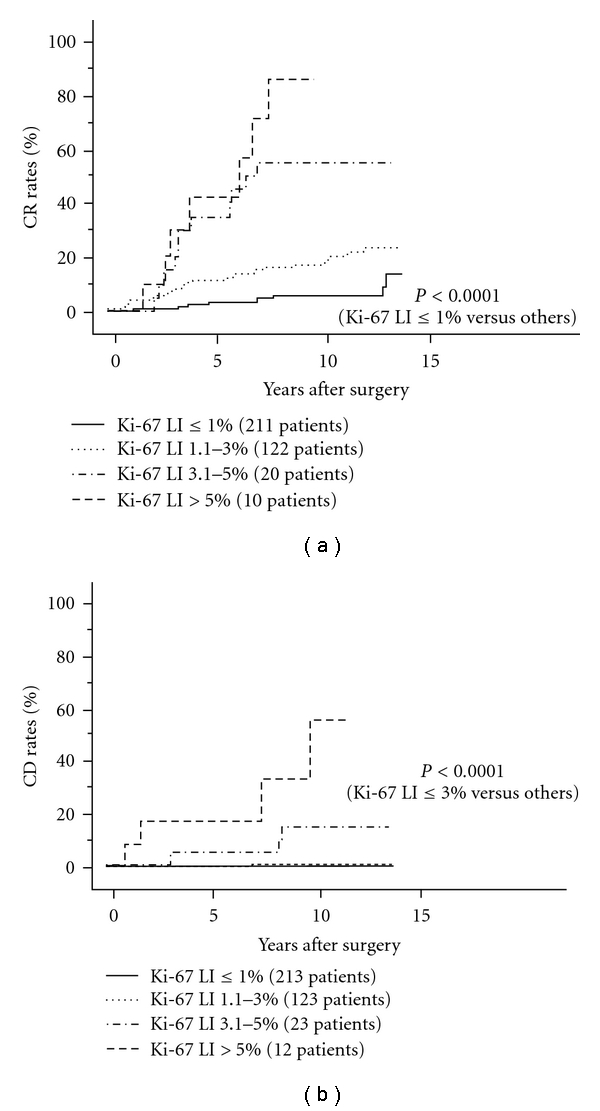
CR rates of PTC patients with Ki-67 1% or less, 1.1–3%, 3.1–5%, and greater than 5%.

 Therefore, it is concluded that Ki-67 LI is the most useful marker for evaluation of cell proliferating activity for PTC. Interpretation of Ki-67 LI data varies according to the origin of carcinoma, but counting Ki-67 LI in hot spots is generally accepted for PTC, because Ki-67 LI in PTC is mostly low. Previous reports examined Ki-67 LI in specimens obtained from FNAB and found that Ki-67 LI ≥ 4% predicted a worse CSS [96]. However, FNAB specimens are not always collected from hot spots, indicating that FNAB specimens with low Ki-67 LI do not always reflect low proliferating activity of PTC tissue. In our previous study, Ki-67 LI directly related to extrathyroid extension, patient age, and distant metastasis at surgery [97]. Furthermore, Ki-67 LI > 1% and >3% were independent prognostic factors for DFS and CSS, respectively, (Figures 9(a) and 9(b)). The optimal cutoff of LI should be determined in each institution, but PTC with high Ki-67 LI should be carefully followed.

#### 2.4.5. *BRAF *Gene Mutation

BRAF is a Raf kinase and a potent activator of the MAPK pathway contributing largely to cell proliferation, apoptosis, survival, and tumorigenesis [98–100]. *BRAF* mutation has been detected in various human malignancies and the hot-spot mutation is a thymine-to-adenine transversion at nucleotide 1799 (T1799A) on exon 15, resulting in a valine-to-glutamate substitution at residue 600 (V600E) [101]. In thyroid carcinoma, studies for *BRAF* mutation have been actively performed. This event was exclusively detected in PTC and its prevalence ranged from 28% to 83% [102–109]. Reports from Western countries and some Asian countries demonstrated that the prognosis of PTC with *BRAF* mutation was significantly worse than that with no mutation [102, 103, 105–109]. 

 In our data analyzing 631 patients, however, *BRAF* mutation was not linked to any clinicopathological features of PTC patients or affected prognosis of patients even on univariate analysis [110]. Its prevalence varies according to histology, and it was higher in tall cell variant and lower in follicular variant than in conventional PTC, which was not discrepant with previous studies [111, 112]. 

 Although there seems to be a consensus that *BRAF* mutation significantly reflects an aggressive character in PTC in Western countries and some Asian countries, its role remains unclear in Japan, because there have not been any other studies in a large number of patients with long-term followup, to date.

### 2.5. Discussion and Summary

Due to the prevalence of ultrasonography-guided FNAB, most PTC can be diagnosed before surgery. There are several important prognostic factors for PTC based on patient backgrounds and findings on preoperative imaging studies. Furthermore, some prognostic factors are evaluated based on intraoperative findings. Therefore, the biological behavior, including prognosis of patients, of PTC can generally be evaluated before the end of surgery. Pathological evaluation is less significant than preoperative and intraoperative evaluations, but it is still important to diagnose certain variants and the involvement of PDC components, both of which affect patient prognoses. 

 Based on the series in our study, the important prognostic factors of PTC patients are age 55 years or older, distant metastasis at surgery, clinical lymph-node metastasis measuring 3 cm or larger, extranodal tumor extension, and significant extrathyroid extension (especially for large PTC, e.g., larger than 2 cm). Tumor larger than 4 cm, clinical node metastasis smaller than 3 cm with no extranodal tumor extension, and male gender are moderate prognostic factors. Latent node metastasis detected only on pathological examination and multicentricity can be prognostic factors but the impact of these is low. 

 Apart from the above, there are some important pathological findings affecting prognosis. Tall cell variant, columnar cell variant, and involvement of PDC components in the majority of the carcinoma lesion are significant prognostic factors on pathological examination. High Ki-67 LI might be a predictor of a dire prognosis. Presence of limited PDC components is also a moderate to weak prognostic factor, because it predicts a worse DFS but not CSS.  Table 2 summarizes the risk factors of PTC based on preoperative, intraoperative and pathological evaluations.

## 3. Prognostic Factors of FTC

FTC is only occasionally diagnosed on preoperative cytology, which is in sharp contrast to PTC. Most patients with FTC undergo hemithyroidectomy based on the preoperative diagnosis as follicular tumor or adenomatous nodules. Generally, patients are diagnosed as having FTC on postoperative pathological examination. FTC is less likely to metastasize to the regional lymph node and lymph-node dissection is rarely performed in surgery for patients suspected of FTC. Extrathyroid extension is also a rare event and in our series, only 2% of patients showed an extension to adjacent organs [113]. Therefore, prognostic factors of FTC based on preoperative findings were less significant than those of PTC. 

### 3.1. Prognostic Factors Based on Patient Backgrounds

#### 3.1.1. Patient Age

Patient age has been adopted as a significant prognostic factor for FTC [114–119]. In our series of 334 patients, age 45 years or older had a prognostic impact for CSS on univariate analysis but was not an independent prognostic factor [113]. Interestingly, in contrast to PTC, age cutoff at 45 years had the strongest prognostic impact in our series.

#### 3.1.2. Gender

**Figure 10:**
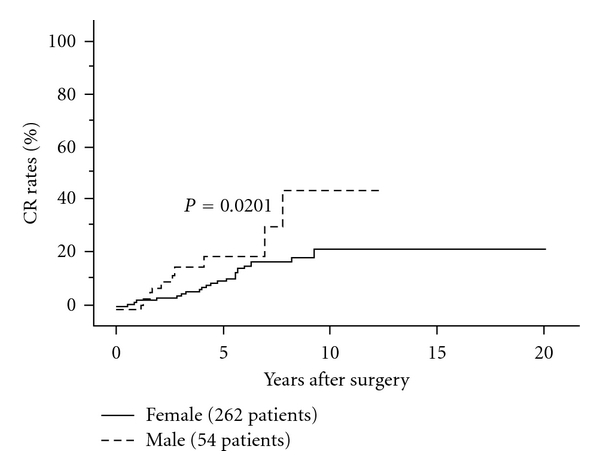
CR rates of female and male FTC patients.

Some previous studies demonstrated the prognostic impact of gender of FTC patients [16, 119, 120]. In our series, male patients tended to show recurrence only on univariate analysis (Figure 10). However, male gender did not show any prognostic impact on CSS of patients.

### 3.2. Prognostic Factors Based on Preoperative Evaluation

#### 3.2.1. Clinical Lymph-Node Metastasis

Since it is rare for FTC to show clinically apparent node metastasis, it is difficult to evaluate its prognostic value. However, as indicated below, we demonstrated that 10 of 11 patients showing pathological node metastasis showed an aggressive pathology such as PDC or widely invasive carcinoma [113], indicating that FTC with clinically apparent node metastasis is progressive and shows an aggressive behavior.

#### 3.2.2. Distant Metastasis at Surgery

Undoubtedly, this is an important prognostic factor as shown in previous studies including ours [16, 113–122]. Five-year CSS of patients with distant metastasis at surgery was around 55% in our series [113]. Distant metastasis at surgery was directly linked to age 45 years or older, lymph-node metastasis, invasive grade, and poor differentiation in our series. For CSS, distant metastasis at surgery or locally palliative surgery was the most important prognostic factor on multivariate analysis.

#### 3.2.3. Tumor Size

Relationship between tumor size and prognosis has been reported [120, 121]. We previously demonstrated that tumor larger than 4 cm affected both DFS and CSS patients on univariate analysis. On multivariate analysis, tumor size had a marginal significance for DFS, but not for CSS.

#### 3.2.4. Family History

Familial FTC accounted for 1.4% of FTC in Japan, but prognosis of these cases did not differ from that of FTC with no family history [123]. To date, there have not been any other reports regarding this issue.

### 3.3. Prognostic Factors Based on Intraoperative Findings

#### 3.3.1. Extrathyroid Extension

As indicated above, extrathyroid extension is a rare event for FTC in Japan. In our series, all patients who showed extrathyroid extension were diagnosed as having PDC components [113]. It is, therefore,suggested that extrathyroid extension is an unusual event for FTC but predicts an adverse prognosis.

### 3.4. Prognostic Factors Based on Pathological Examination

#### 3.4.1. Degree of Invasiveness

**Figure 11:**
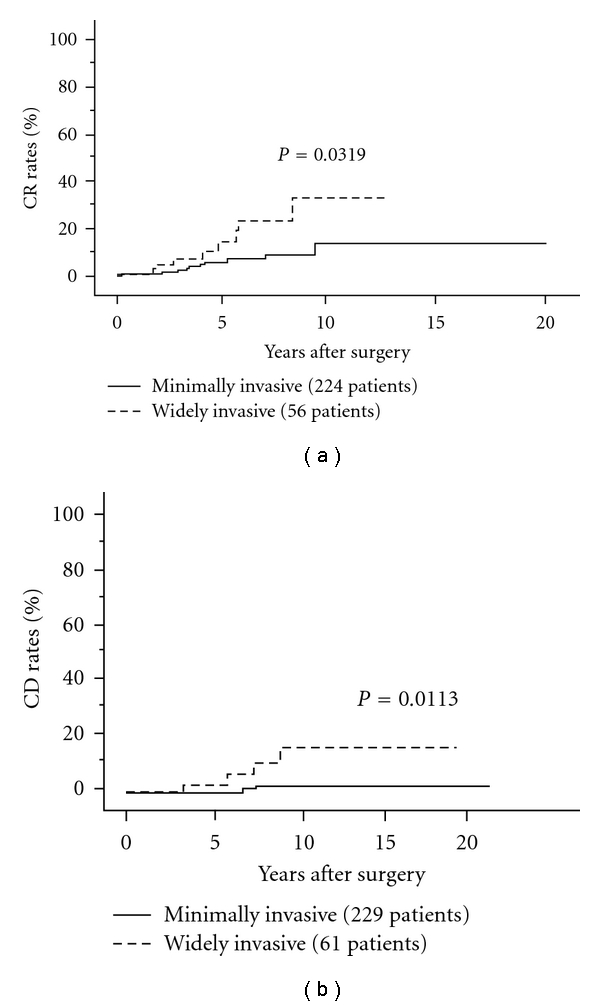
CR rates of minimally and widely invasive FTC.

FTC is pathologically diagnosed when it shows capsular and/or vascular invasion of tumor cells. FTC is divided into two categories: (1) minimally invasive FTC, small number of invasive sites can be only histologically detected and (2) widely invasive FTC, invasive sites, and/or vascular invasion are extensively present and even grossly detected. Previous studies demonstrated that widely invasive FTC was more likely to show recurrence and had a poorer prognosis than minimally invasive FTC [113, 124, 125]. Especially, extensive vascular invasion significantly affects prognosis of FTC. Therefore, it is very important to diagnose whether an FTC is widely or minimally invasive on pathological examination to predict patient prognosis and select the subsequent therapy. In our series, carcinoma recurrence rates and carcinoma death rates of patients with widely invasive FTC were significantly greater than those of patients with minimally invasive FTC [113] (Figures 11(a) and 11(b)). Since most FTC patients underwent hemithyroidectomy in initial surgery, we recommend completion total thyroidectomy followed by RAI ablation for widely invasive FTC patients but not for minimally invasive FTC patients unless no distant metastases were detected.

#### 3.4.2. Poor Differentiation

**Figure 12:**
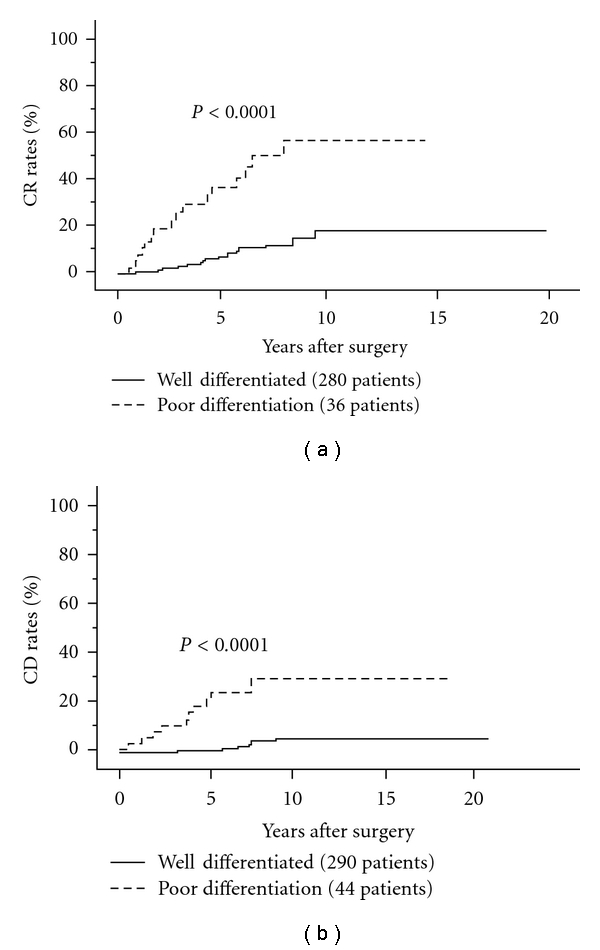
CR rates of FTC patients with and without PDC components.

Involvement of PDC component is a more significant prognostic factor for FTC than that for PTC. In our experience, PDC arising from FTC is generally diagnosed as PDC on WHO classification, because of the presence of PDC component in the majority of the tumor. In our series, 5-year and 10-year carcinoma recurrence rates of FTC having PDC component were 37% and 57%, and its carcinoma death rates were 21% and 29%, respectively [113] (Figures 12(a) and 12(b)). On multivariate analysis, this is the strongest prognostic factor for DFS. For CSS, this had the second strongest prognostic impact after distant metastasis at surgery.

#### 3.4.3. Oxyphilic Cell Variant

Oxyphilic cell variant is a particular type of FTC on WHO classification. Wide cytoplasms with eosinophilic granules and clear nuclear bodies are typical findings on hematoxylin and eosin-stained tissue sections. One study showed that most cases of oxyphilic cell variant were RAI refractory [126]. In Europe and the United States, many researchers have demonstrated a worse prognosis of oxyphilic cell variant than conventional FTC [116, 126, 127]. However, there are studies demonstrating that prognosis of oxyphilic cell variant does not differ from [128, 129] or was even better than that of conventional FTC [130]. In Japan, there are two studies investigating the prognosis of oxyphilic cell variant and both demonstrated that the prognosis of oxyphilic cell variant was similar to that of conventional FTC [113, 131]. It is, therefore, not evident that oxyphilic cell variant shows an aggressive behavior in Japan. The prevalence of oxyphilic cell variant was 13% in our series [113], which is similar to that in the United States [116].

#### 3.4.4. Pathological Lymph-Node Metastasis

Lymph-node dissection is not performed for surgery for patients unless metastatic nodes are clinically detected or cytological examination suggests high-grade malignancy such as PDC. In our series, 11 of 334 patients (3%) had pathologically diagnosed lymph-node metastasis and 10 of these patients were diagnosed as having widely invasive FTC or PDC as indicated above [113]. During postoperative followup, 10 patients (3%) showed recurrence to the regional lymph nodes after surgery and 8 of these were widely invasive carcinoma or included PDC components in specimens in initial surgery. Therefore, although rare, the presence of lymph-node metastasis reflects aggressive behavior of FTC. A report form Germany also demonstrated the prognostic impact of lymph-node metastasis of FTC and recommended extensive lymph-node dissection, including MND, for FTC demonstrating a large tumor and/or extrathyroid extension [120].

### 3.5. Discussion and Summary

In contrast to PTC, FTC can rarely be diagnosed on preoperative examinations, including ultrasonography and cytology. The most significant prognostic factor was the presence of distant metastasis at surgery. Apart from that, pathological findings such as the involvement of PDC components and widely invasive lesions were significant prognostic factors. Although oxyphilic cell variant did not affect prognosis in studies from Japan, many studies from Western countries indicated that this variant is a sign of aggressive behavior. Taken together, pathological evaluation of FTC is very important to predict patient prognosis and select the therapeutic strategy such as whether completion total thyroidectomy should be performed. Patient age, gender, and tumor size have a moderate prognostic impact. Table 3 summarizes the risk classification of FTC. In addition, extrathyroid extension and pathological lymph node metastasis are rare events in FTC, but these findings are much more likely detected in patients showing an aggressive histology. Although these findings can be signs of aggressive behavior, it remains unclear whether they are independent prognostic factors.

## 4. Conclusions

The significance of preoperative evaluation and pathological examination differs between PTC and FTC. For PTC, preoperative findings on imaging studies and intraoperative findings are quite important to evaluate biological behavior, including prognosis, and therapeutic strategies can be established for most patients at that time. Distant metastasis at surgery, extrathyroid extension, large lymph-node metastasis, and extranodal tumor extension are significant prognostic factors and extensive surgery and adjuvant therapy with careful followup are mandatory for patients with such characteristics.

 In contrast, postoperative pathological examination has a significant value not only for diagnosis of FTC but also for evaluating biological characteristics. Similar to PTC, distant metastasis at surgery is the strongest prognostic factor, but pathological findings such as grade of invasiveness and carcinoma differentiation are also very important to evaluate its biological behavior. Such pathological findings are essential to decide whether to perform further therapies such as completion total thyroidectomy with RAI therapy for FTC.

## Figures and Tables

**Table 1:** Prevalence of conventional and various histologic variants in 1521 papillary carcinomas.

Conventional	86%
Follicular variant	7%
Tall cell variant	4%
Oncocytic variant	2%
Columnar cell variant	0.4%
Macrofollicular variant	0.3%
Diffuse sclerosing variant	0.3%
Cribriform morular variant	0.1%
Others	0.1%

**Table 2:** Risk classification of TPC based on preoperative, intraoperative, and pathological findings.

High risk
(a) Patients having distant metastasis at presentation.
(b) Patients including PDC components in majority of carcinoma lesions.
(c) Patients diagnosed as tall cell variant.
(d) Patients 55 yrs or older and having one of the following three:
(1) tumor size >2 cm with extension to adjacent organs, (corresponding to T4),
(2) lymph-node metastasis >3 cm,
(3) lymph-node metastasis extending to adjacent organs

Intermediate risk
(a) Patients including PDC component in small portion of carcinoma lesions.
(b) Patients 55 yrs or older and having one of the following three:
(1) tumor size >4 cm (excluding T4 cases),
(2) tumor size ≤2 cm with extension to adjacent organs (corresponding to T4),
(3) lymph-node metastasis detectable on preoperative imaging studies ≤3 cm and without extension to adjacent organs

Low risk
(a) Patients under 55 yrs without distant metastasis at diagnosis.
(b) Patients 55 yrs or older who are not classified as high risk or intermediate risk

**Table 3:** Risk classification of FTC based on preoperative, intraoperative, and pathological findings.

High risk
Patients having one of the following three:
(1) patients having PDC components in the majority of carcinoma lesions,
(2) patients diagnosed with widely invasive carcinoma (extensive capsular or vascular invasion),
(3) patients having distant metastasis at diagnosis.

Intermediate risk
Patients without high-risk characteristics and having one of the following two:
(1) patients 45 yrs or older,
(2) patients having tumor >4 cm.

Low risk
Patients who are not classified as high-risk or intermediate risk

Patients with extrathyroid extension and lymph-node metastasis may be classified into high and intermediate risk, respectively.
